# Primary dedifferentiated liposarcoma of the ovary: expanding the surgical management spectrum with a different resection extent than existing reports – a case report and literature review

**DOI:** 10.3389/fonc.2025.1665723

**Published:** 2025-12-10

**Authors:** Hao Liang, Mingxia Wen, Xuzhi Liang, Zili Lv, Jiangtao Fan

**Affiliations:** 1 Department of Gynecology and Obstetrics, The First Affiliated Hospital of Guangxi Medical University, Nanning, Guangxi, China; 2 Department of Pathology, The First Affiliated Hospital of Guangxi Medical University, Nanning, Guangxi, China

**Keywords:** liposarcoma, dedifferentiated, ovarian neoplasms, surgery, operative therapy

## Abstract

**Background:**

Dedifferentiated liposarcoma (DDLPS) is a relatively common type of liposarcoma, typically occurring in the retroperitoneum and limbs. It is rarely found in the female reproductive system, leading to the absence of a standard treatment protocol for primary ovarian dedifferentiated liposarcoma. This case presents a personal experience-based approach, as there is a lack of standard treatment guidelines, relying on treatment consensus for more common locations and decades of clinical experience.

**Case presentation:**

This case report describes a 55-year-old woman who was admitted due to a gradually enlarging mass in the right lower abdomen, accompanied by bloating and abdominal pain. Physical examination revealed a palpable abdominal mass. Gynecological ultrasound showed a hyperechoic mass measuring 135*128*91mm in the pelvic-abdominal cavity. CT imaging suggested a large cystic and solid mixed liposarcoma originating from the ovary, with unclear delineation of the uterine fundus and slight compression of surrounding organs. Pelvic MRI revealed an irregular mass approximately 10.7cm×12.6cm×12.2cm, with slightly prolonged T1 and heterogeneous T2 signals, showing fat signal in the right fatty tissue, suggesting invasion of the uterine wall by liposarcoma. The patient then underwent open surgery, and postoperative pathology with immunohistochemistry confirmed dedifferentiated liposarcoma. FISH testing was positive for MDM2, confirming the diagnosis of ovarian-origin dedifferentiated liposarcoma. Although the patient refused chemotherapy, she has been followed up every three months, and the current follow-up shows no signs of tumor recurrence.

**Conclusion:**

Ovarian dedifferentiated liposarcoma is an extremely rare condition, with surgery being the preferred treatment method. This report presents a rare case of ovarian dedifferentiated liposarcoma, providing a reference for clinical diagnosis and treatment. Through collaboration between gynecology, pathology, and imaging departments, the accuracy of the diagnosis and comprehensiveness of treatment were ensured. Long-term follow-up of the patient provided valuable insights into the recurrence and prognosis of the disease.

## 1 Introduction

Liposarcoma (LPS) is a relatively rare mesenchymal tumor in clinical practice, with the most common histological types including well-differentiated, dedifferentiated, and myxoid variants. These tumors can occur in all parts of the body, most commonly in the limbs, trunk, and retroperitoneal cavity, though very few cases are reported in the female reproductive system. Only dedifferentiated liposarcoma (DDLPS) is commonly associated with high-grade liposarcoma (WDLPS), accounting for nearly 60% of LPS cases. DDLPS, as one of the most common subtypes of liposarcoma, has an annual incidence rate of 0.21 cases per 100,000 persons, with fewer than 2,500 new cases in the United States each year (1). To date, there have been fewer than 20 reported cases of primary ovarian DDLPS worldwide. Although some literature has reported cases of ovarian dedifferentiated liposarcoma (2), this case not only clearly defines the gold standard for diagnosing ovarian DDLPS but also highlights a significantly different surgical approach compared to previous reports. The prognosis of DDLPS is particularly influenced by the anatomical site of onset, so surgical resection should be tailored based on the tumor’s location. For rare locations like primary ovarian DDLPS, special attention must also be given to the details of surgical decision-making.

Although DDLPS is often closely related to WDLPS, with some literature suggesting that it typically progresses from WDLPS into a non-lipogenic sarcoma (3), the pathological transformation between WDLPS and DDLPS is often abrupt. In some excised specimens, well-differentiated adipocyte components can be difficult to identify. Histopathological features of both WDLPS and DDLPS can coexist within the same tumor mass. DDLPS lacks a distinctive histological appearance and exhibits a wide morphological spectrum, ranging from classic pleomorphic features to relatively uniform spindle or epithelial-like cells, making it easy to confuse with other soft tissue sarcomas (4, 5). Although the incidence of DDLPS is lower than that of WDLPS, DDLPS progresses more rapidly, has higher malignancy, and is more likely to metastasize and recur. The clinical presentation in the early stages is also nonspecific, which makes pathological examination, immunohistochemical staining, and FISH testing crucial for diagnosis. Therefore, it is essential for pathologists to maintain a high level of suspicion when dealing with suspected DDLPS cases.

Treatment options typically include surgery, chemotherapy, and radiotherapy, while the efficacy of targeted therapy and immunotherapy remains to be further studied. Treatment regimens for DDLPS of the limbs and retroperitoneal cavity have been well-established, but due to the rarity of ovarian DDLPS, there is currently no standardized treatment protocol. As mentioned in several case reports, primary ovarian DDLPS is extremely rare, and due to the lack of characteristic symptoms and laboratory findings, it can be difficult to confirm the nature of the mass. Although ultrasound and imaging studies may suggest a pelvic location, past reports (2) indicate that because of the rare occurrence and some cases (6) originating from the mesentery or sigmoid colon mesentery, it is easy to misinterpret such cases as originating from the mesentery but developing in the ovary. Therefore, accurate diagnosis still requires surgical and pathological confirmation.

This report describes a case of ovarian dedifferentiated liposarcoma treated with surgery, which was successfully resected with no recurrence and significant clinical benefit. Furthermore, a retrospective analysis of relevant research on ovarian dedifferentiated liposarcoma over the past 20 years was conducted through databases such as PubMed and GeenMedical. The analysis explores surgical approaches, personalized treatment plans, and the pathogenesis of this rare tumor in order to improve patient prognosis.

## 2 Case report

**Figure 1:**
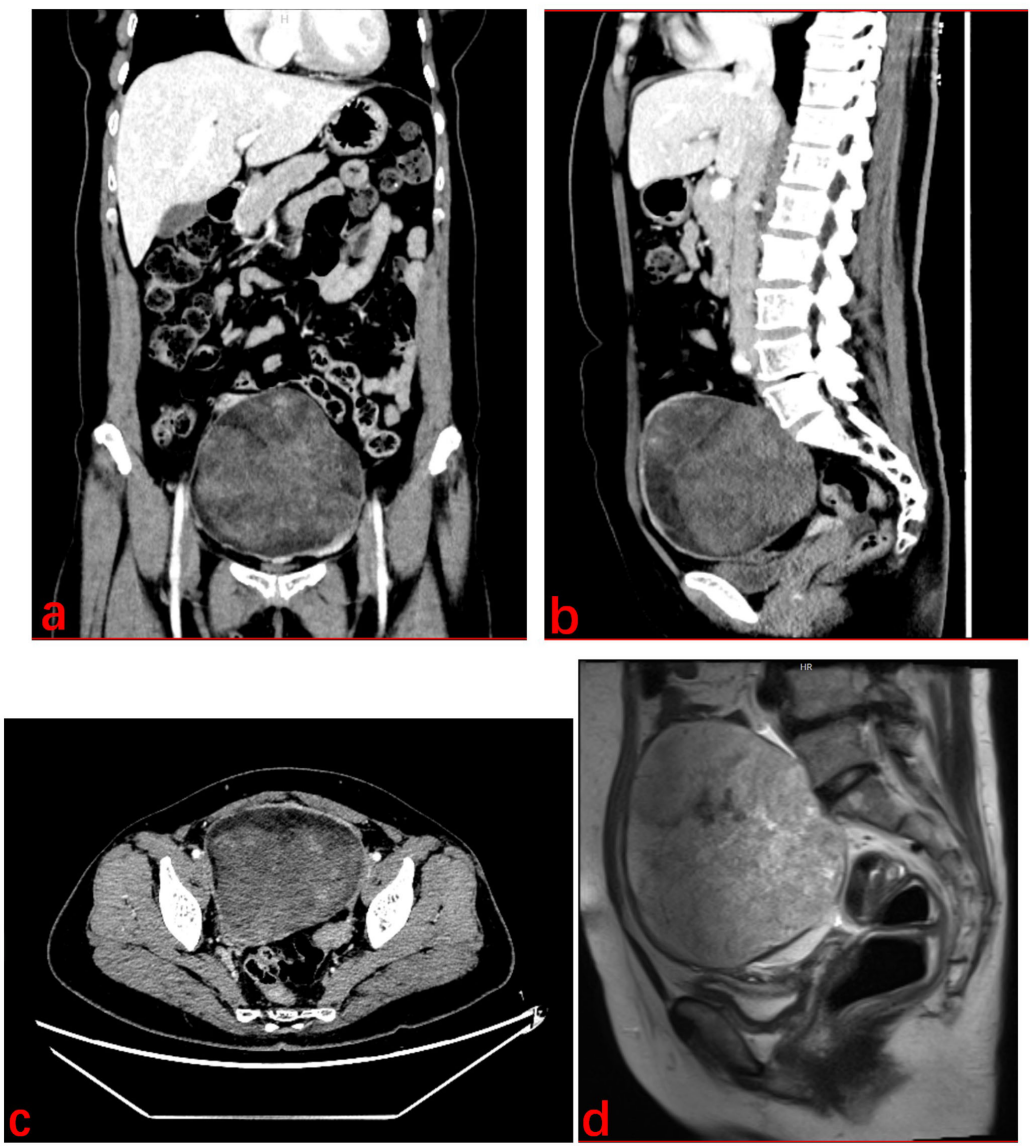
Abdominal CT. **(A)** Coronal section; **(B)** Sagittal section. **(C)** Cross section. **(D)** MRI of abdominopelvic mass.

**Figure 2:**
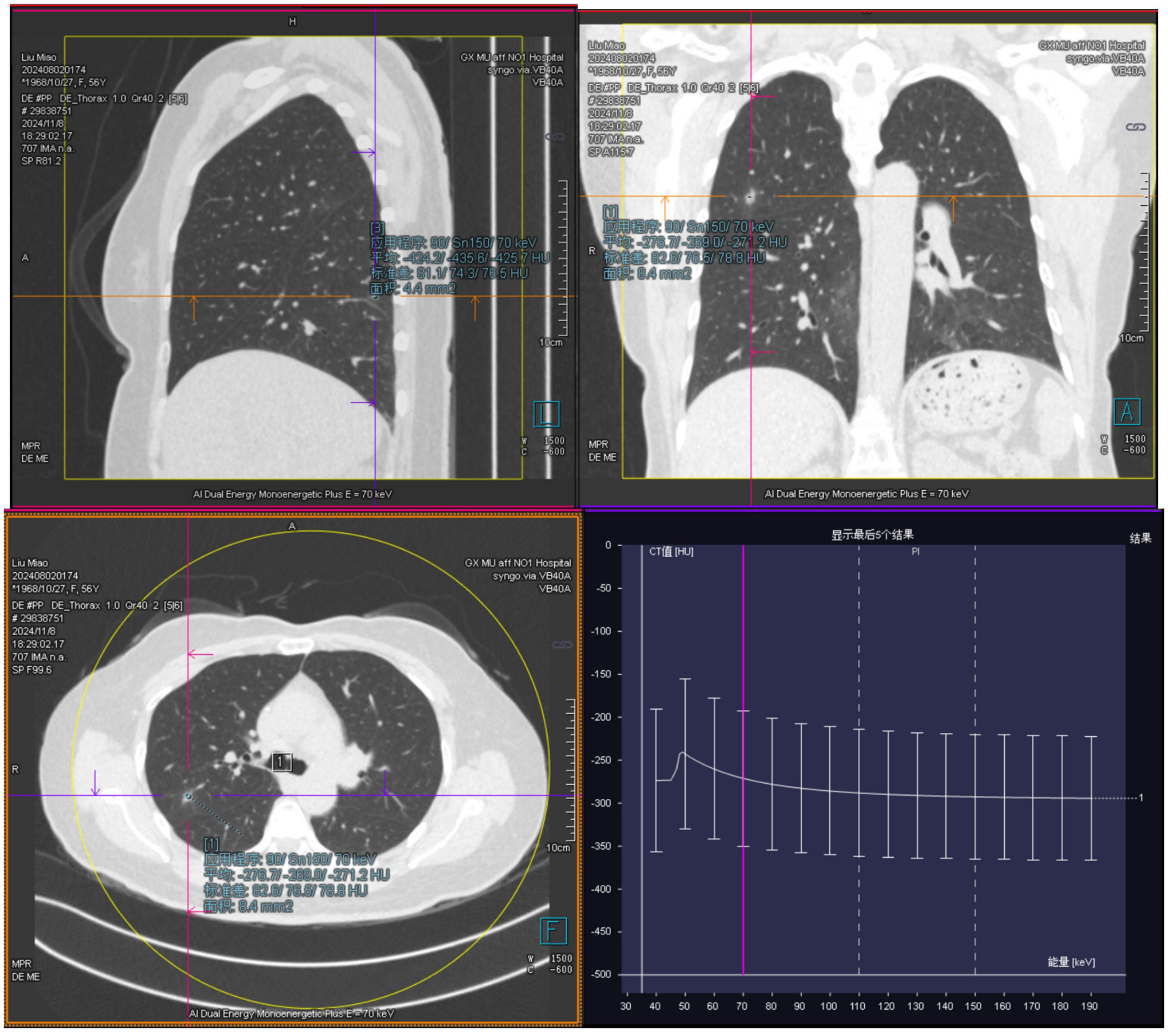
Ground-glass nodules are visible in the lungs on chest computed tomography (CT), with a close relationship to the surrounding blood vessels.

55-year-old female patient presented with a movable, painless mass the size of a quail egg in the right lower abdomen, which she first noticed in March 2024. She sought medical attention on August 5, 2024, due to an increase in size of the mass, abdominal bloating, and occasional dull pain in the right lower abdomen. The patient had been postmenopausal and had a history of two induced abortions and one cesarean section, with no other postmenopausal bleeding. There was no significant medical history, and no family history or genetic predisposition to malignant tumors. Clinical examination revealed a palpable abdominal mass. Gynecological ultrasound showed a hyperechoic mass in the pelvic and abdominal cavity, measuring 135*128*91mm. Doppler ultrasound revealed dotted and rod-shaped blood flow signals within the mass, suggesting a possible liposarcoma. CT (**Figures 1A–C**) showed a large cystic-solid mixed lesion, approximately 10.7cm×12.0cm×11.7cm in size, with clear margins and internal septations, displaying cystic and solid mixed characteristics. The minimum value was -61 Hu, with unclear demarcation of the uterine fundus and slight compression of adjacent organs. The lesion was suspected to be a liposarcoma of ovarian origin that has not been excluded. Additionally, lung CT (**Figure 2**) revealed mixed ground-glass nodules in the posterior segment of the right upper lobe, the posterior basal segment of the right lower lobe, and pure ground-glass nodules in the apical posterior segment of the left upper lobe, which were closely related to surrounding blood vessels, suggesting a possible tumor. MRI (**Figure 1D**) showed an uneven, slightly prolonged T1 and mixed T2 signal mass measuring approximately 10.7cm×12.6cm×12.2cm. Fat tissue signals were observed on T2 fat suppression, suggesting liposarcoma invasion of the uterine wall. Therefore, imaging results suggested liposarcoma in the pelvic and abdominal cavity, but the exact origin, whether ovarian or not, remained unclear. The ground-glass nodules in the lungs raised concern, but it was difficult to determine if they were incidental or metastatic based solely on the chest CT. Her sex hormones and tumor markers, including coagulation function, were all normal.

**Figure 3:**
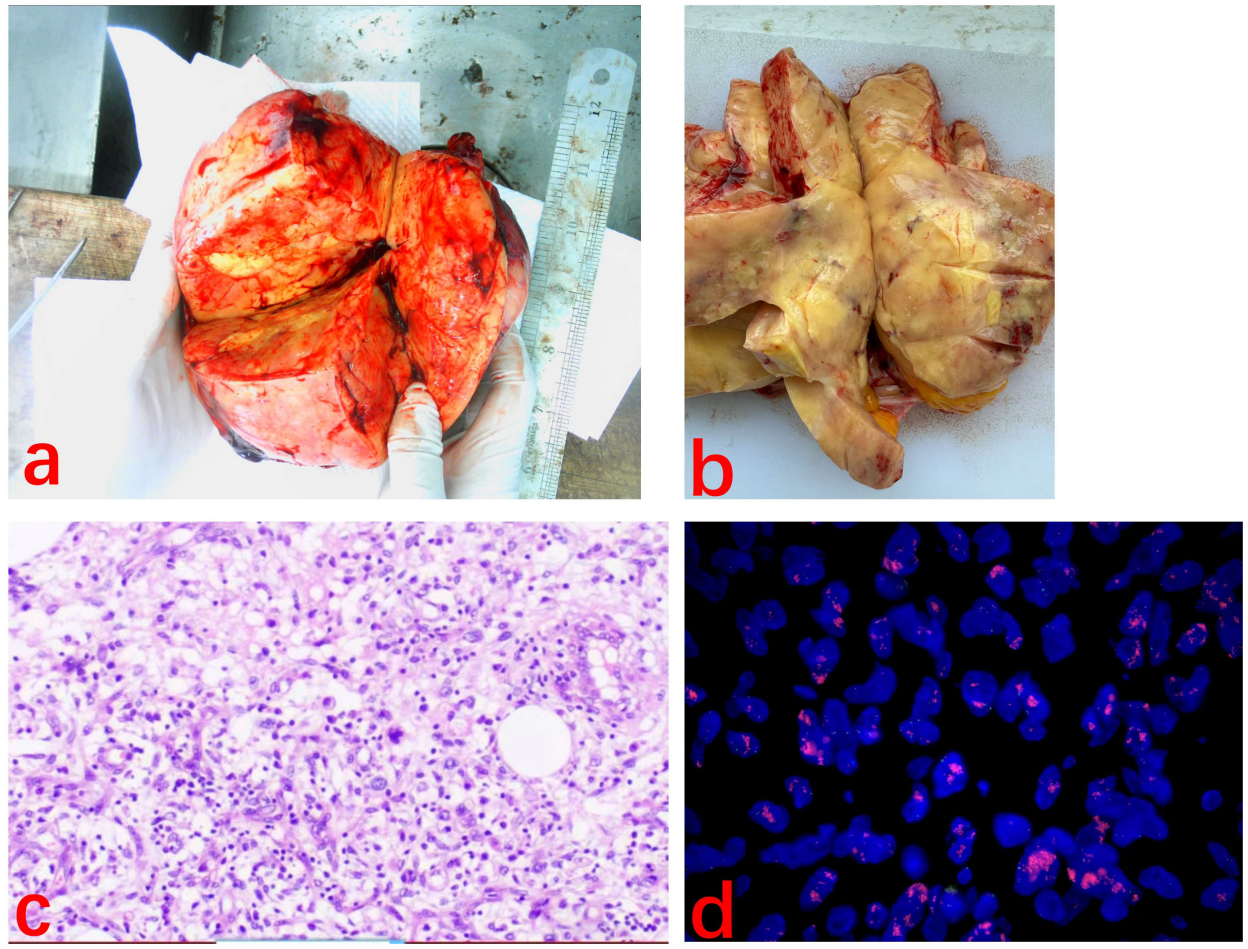
**(A, B)** Macroscopic appearance of the part of tumor. The tumor appeared yellow and solid. **(C)** Spindle-shaped cells are diffusely arranged with scattered adipocyte-like features. (H&E ×100). **(D)** MDM2 FISH slide. Break signals are easily detected in the translocated cell.

**Figure 4:**
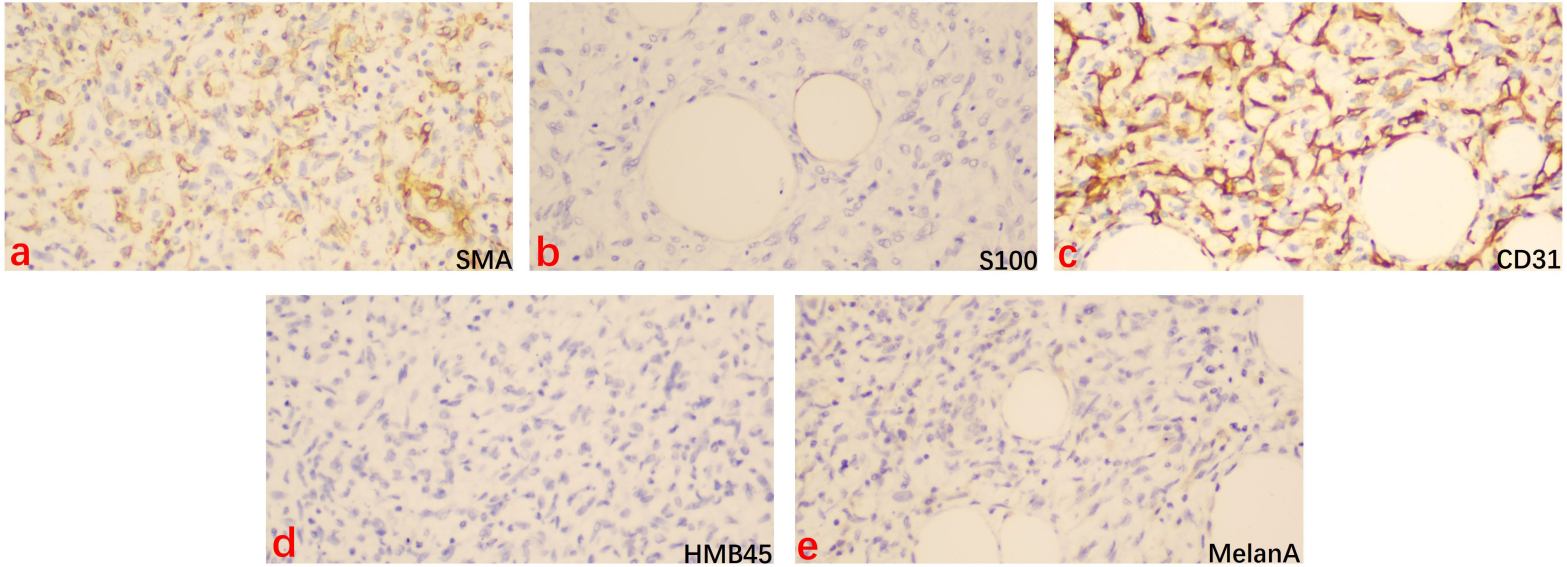
Immunohistochemical staining for SMA, S100, CD31, HMB45, and MelanA in the dedifferentiated liposarcoma tissue (×200). Brown staining indicated the positive cells. **(a)** SMA = smooth muscular actin, **(b)** S100 = S100 calcium binding protein B, **(c)** CD31 = Platelet And Endothelial Cell Adhesion Molecule 1, **(d)** HMB45 = Melanosome, **(e)** MelanA = Melanoma antigen recogmized by T cell-1.

After MDT discussion, considering that surgical treatment is the cornerstone and the first choice for liposarcoma (1), the final surgical plan was determined on August 9, 2024, to perform an open surgery following the radical surgery approach for ovarian cancer, including total abdominal hysterectomy with bilateral adnexectomy, pelvic lymph node dissection, aortic lymph node dissection, and omentectomy. After excision, the right ovary was found to be a large spherical tumor with an intact capsule. Upon opening, the tumor was yellow and solid, and pathology revealed diffuse spindle cell distribution, interspersed with mature adipocytes, without signs of hemorrhage or necrosis, and few mitotic figures (**Figures 3A–C**). Immunohistochemistry results (**Figure 4**) were negative for HMB-45, Melan A, S-100, CK, and CD31, but positive for Vimentin, SMA, and CD34, indicating dedifferentiated liposarcoma. FISH testing (**Figure 3D**) showed MDM2 positivity. No lymph node metastasis was observed. Based on the FIGO staging, the diagnosis was ovarian dedifferentiated liposarcoma stage IA. After confirming the diagnosis of DDLPS with MDM2 amplification, we recommended chemotherapy for the patient. Unfortunately, the patient refused further chemotherapy and insisted on self-treatment with traditional Chinese medicine at an external hospital. Four months after discharge, with no abnormalities or recurrence in the pelvis, the lung ground-glass nodules were still undetermined in terms of metastasis. The patient underwent laparoscopic left upper lobectomy and right lower lobectomy with lymph node dissection in the thoracic surgery department. Pathology showed tumor wall-adherent growth, which was suggestive of carcinoma *in situ*. The patient was followed up every three months after discharge, and as of September 2025, the abdominal bloating symptoms had completely resolved, and no abnormalities were found in the pelvis. We will continue to closely monitor the patient.

## 3 Discussion

DDLPS typically presents clinically as a painless mass that gradually increases in size, with symptoms often related to the location of the mass (7). In an analysis of the NCDB database, it was found that although the most common primary site for DDLPS is the retroperitoneum or abdomen (59.5%), the most fatal cases are also in the retroperitoneum or abdomen, with the reason closely related to tumor size (tumors smaller than 10 cm have better 5-year and 10-year survival rates) (8). While DDLPS is a relatively common, aggressive, high-grade soft tissue sarcoma with a high local recurrence and distant metastasis rate (9), primary ovarian DDLPS is rare, and it is often reported as a case study in the literature. Regarding its pathogenesis, some studies suggest that DDLPS shares characteristic genomics similar to ALT/WDLPS, originating from an excessive ring chromosome or giant chromosome on the long arm of chromosome 12 (12q), containing a highly amplified 12q14–15 region, including the MDM2 gene located at 12q15 (10). The MDM2 gene is a key driver gene in the development of ALT/WDLPS and DDLPS tumors. Its stable and sustained amplification and overexpression are among the earliest events in liposarcoma formation (11). The protein encoded by MDM2 negatively regulates the tumor suppressor gene p53 by inhibiting nuclear transcription and replication, as well as enhancing protein degradation. Additionally, MDM2 gene amplification further affects p53 protein’s apoptotic function, promoting tumor proliferation (12, 13). Therefore, using fluorescence *in situ* hybridization (FISH) to detect MDM2 gene amplification is highly specific and sensitive for diagnosing DDLPS (in the appropriate histological context) (14). In this case, pathology suggested diffuse spindle cells accompanied by mature adipocytes, with rare mitotic figures and no hemorrhage or necrosis, and CD34 showed varying degrees of positivity in immunohistochemistry. Therefore, when distinguishing from atypical spindle cell lipoma-like tumors, FISH detection of MDM2 amplification becomes the gold standard for diagnosing DDLPS (15). After combining clinical examination, immunohistochemistry, and molecular testing results, we quickly diagnosed this patient with primary ovarian dedifferentiated liposarcoma. The supraphysiological level of MDM2 in DDLPS widely affects carcinogenic transcriptional circuits. Therefore, in the future, MDM2 inhibitors may be a reasonable treatment strategy for DDLPS (16). However, preclinical studies have shown that due to the heterogeneity of MDM2 copy numbers within tumors, cells with extremely high copy numbers may be resistant to MDM2 inhibition. Additionally, MDM2 inhibition causes an initial surge in p53 levels, which further drives MDM2 expression (17, 18). Therefore, although there is no clear mechanism of resistance, these research findings provide a theoretical reference for further understanding of resistance mechanisms. It is crucial to further develop predictive biomarkers to assist patients in making treatment decisions (19).

Ideally, the diagnosis of DDLPS should include cross-sectional imaging of the primary tumor, evaluation of potential metastases, and pathological assessment, based on pre-treatment biopsy. However, modern imaging techniques have advanced significantly. For DDLPS in the limbs or trunk, MRI is the preferred method for evaluating the primary tumor as it provides the best preoperative assessment of the tumor’s relationship with adjacent soft tissue, neurovascular structures, and the extent of involvement. For DDLPS in the retroperitoneum or abdominal cavity, enhanced CT is the preferred imaging method. The relationship between the tumor and major vascular structures determines the resectability. However, preoperative imaging evaluation for ovarian liposarcoma is rarely mentioned. In this case, the patient initially sought gynecological consultation due to discomfort. Routine gynecological ultrasound suggested a pelvic mass suspicious for liposarcoma, and tumor markers were normal. Subsequently, pelvic MRI and enhanced CT of the chest, abdomen, and pelvis were performed, indicating a suspected ovarian liposarcoma with uterine wall involvement. Additionally, a ground-glass nodule was found in the lungs, which was later confirmed as primary lung carcinoma. Numerous studies have mentioned that DDLPS is prone to lung metastasis (20). Moreover, some studies suggest that due to the special overexpressed genomics in DDLPS, these genes could potentially be responsible for secondary cancers, and because liposarcomas are often large, they can trigger systemic immune responses, making patients susceptible to second malignancies (21). Therefore, lung CT should be added during treatment to rule out metastasis and secondary malignancies.

Due to the lack of unique histological features of DDLPS, we should also differentiate it from other types of liposarcoma in the diagnosis of ovarian dedifferentiated liposarcoma. This case should be distinguished from primary ovarian smooth muscle sarcoma (POLMS) (22). Similar to DDLPS, POLMS also primarily presents with lower abdominal pain caused by tumor enlargement. Upon careful observation, although this case also exhibits diffuse spindle cell distribution, it differs in that mature adipocytes are scattered throughout. Immunohistochemically, while this case shows SMA positivity, similar to POLMS, it is negative for S100 and CD31, and POLMS does not exhibit MDM2 positivity. These features can help differentiate and exclude POLMS. Moreover, the immunohistochemistry in this case essentially excludes other possible tumor origins, such as epithelial, peripheral nerve, or vascular tumors. Therefore, when comparing with ovarian stromal sarcomas, in addition to using different pathological morphologies and immunohistochemistry for distinction, FISH testing has also become the gold standard for differential pathological diagnosis. For example, FISH testing for DDIT3 gene rearrangement can be used for the diagnosis of primary ovarian low-grade myxoid liposarcoma (23, 24). However, after diagnosing dedifferentiated liposarcoma, determining the primary site of origin becomes a diagnostic challenge. According to previous literature (6), we need to consider the degree of adhesions visible during surgery and the extent of infiltration into surrounding tissues. Given the unique location of the ovaries, careful consideration should be given to whether the tumor originates from retroperitoneal fat tissue, which may require consideration for extended treatment. Therefore, active postoperative follow-up and evaluation are also crucial.

Due to the clinical rarity of ovarian dedifferentiated liposarcoma (DDLPS), most cases are reported individually, and there is a lack of large sample data studies. Therefore, we can only refer to treatment guidelines for localized DDLPS. The 2021 Transatlantic Australasian RPS Working Group Consensus (25) suggested that the best surgical strategy is to achieve oncologically complete resection during the initial surgery. Ideally, this should include 1–2 cm of normal tissue as margins, and often requires multi-organ resection to ensure macroscopic complete resection. However, this method needs to weigh the short-term and long-term postoperative complications and mortality risks (26). The consensus also excluded solid tumors like gynecological liposarcomas, noting that due to their rare location, there are no established treatment standards (26). Patients with these types of tumors require multidisciplinary discussions and decisions at experienced soft tissue sarcoma treatment centers. Multiple studies have shown that sarcoma patients who are hospitalized more frequently tend to have better prognostic outcomes (27–29).Therefore, combining preoperative imaging results and intraoperative frozen pathology reports, we considered this rare type of ovarian tumor to be aggressive and conducted a multidisciplinary discussion and decision-making process. As previously mentioned in the research conclusions (6–8), dedifferentiated liposarcoma is significantly associated with poor prognosis, and tumor size and location can impact patient survival. Therefore, our center adopted a comprehensive ovarian cancer staging surgery approach in order to achieve complete tumor resection, ensure negative resection margins, and perform macroscopic complete resection while minimizing postoperative recurrence risks. To further ensure the patient’s prognosis, we also presented the case for consultation at Sun Yat-sen University Cancer Center, hoping that the expertise of more experienced sarcoma specialists would lead to better survival outcomes for the patient. Additionally, determining the patient’s postoperative risks helps us oncologists to develop the most suitable personalized treatment plan (30).Postoperatively, with the patient’s FIGO staging at IA and FNCLCC grading at grade 2, but with a rare tumor site, we unanimously recommended chemotherapy for the patient. Unfortunately, the patient refused and is currently undergoing follow-up every three months. In assessing the patient’s postoperative risks, we found that histological grading is one of the factors most closely associated with the prognosis of DDLPS patients. In a 2017 study by Dantey et al., they confirmed that patients with low-grade FNCLCC grading (grade 1) had significantly better survival outcomes than those with higher-grade (grade 2 and 3) tumors (31). However, since the TNM staging system is broad, there is significant heterogeneity among tumors in the same stage, and risk stratification remains imperfect. Additionally, DDLPS is particularly influenced by anatomical location, tumor biological behavior, histological grading, and pathological subtypes (32). The true survival prognostic factors and staging system for DDLPS are still under research, and updated consensus on DDLPS treatment is needed to meet the patient’s needs for better PFS and higher remission/disease control rates (DCR) (33). For example, the 8th edition of the AJCC TNM has provided separate staging algorithms for sarcomas based on anatomical location to address this issue (34). Additionally, due to the low incidence of lymph node metastasis in sarcoma patients (less than 5%), the role of lymph node metastasis (LNM) risk stratification in prognosis models is limited (35). This has also led us to reconsider the decision-making process in ovarian DDLPS treatment, such as whether the range of lymph node dissection should be refined when following ovarian cancer protocols and whether only unilateral adnexal resection can be performed if organ involvement is not indicated preoperatively.

Nevertheless, this study has limitations. Due to the limited number of ovarian DDLPS cases, only one case is reported here, limiting the generalizability and representativeness of the results. Furthermore, the rarity of such cases prevents the establishment of a control group for comparative analysis, further restricting the generalizability of the results. Additionally, the follow-up time is insufficient to comprehensively evaluate long-term prognosis and recurrence rates. Moreover, the treatment plan outlined in this article may not be universally applicable to all similar cases, suggesting that personalized treatment approaches require further study. For example, whether adjuvant treatments such as radiotherapy or chemotherapy, based on grading and staging, would bring clinical benefits to patients needs further multi-center and multi-regional clinical trials.

## 4 Conclusion

This article reports a case of examination and treatment for ovarian stage IA DDLPS and explores the potential application of targeted therapy in reproductive system DDLPS. Primary ovarian liposarcoma is extremely rare in clinical practice and is typically diagnosed through lesion biopsy or postoperative pathology combined with immunohistochemistry and FISH results (e.g., MDM2 positive, CD4 positive, etc.). Due to the rare location of the case, there is no standardized diagnostic or treatment protocol. Treatment usually involves a combination of surgery, radiotherapy, and high-dose multi-drug chemotherapy. In our case, although postoperative chemotherapy was not administered for personal reasons, significant therapeutic effects were achieved through surgery alone. Postoperative survival rates are correlated with the patient’s age, clinical stage, treatment method, histological type, and lesion size. Literature review suggests that histological type and anatomical location profoundly influence the prognosis of DDLPS. Therefore, the grading system needs to be updated and optimized based on anatomical location. Personalized treatments should be provided according to the refined prognoses for different sites of origin. Surgery, as the most fundamental treatment for DDLPS, requires particular attention when it involves special sites like the ovary. The extent of resection has different impacts on the long-term or short-term prognosis of patients. This case report highlights the importance of MDM2 as the gold standard for DDLPS diagnosis, while also offering insights into the surgical scope for this rare location of dedifferentiated liposarcoma. We hope this will provide a perspective for surgical decision-making in future cases of ovarian DDLPS. Additionally, the development of new targeted drugs will play a significant role in advancing the treatment of this disease.

## Data Availability

The original contributions presented in the study are included in the article/supplementary material. Further inquiries can be directed to the corresponding authors.
